# Technical development and user experience evaluation of the Computerised version of the Sydney Test of Activities of daily living in Memory disorders (C-STAM)

**DOI:** 10.3389/fdgth.2025.1637916

**Published:** 2025-11-17

**Authors:** Jeewani Anupama Ginige, Kritika Rana, Marcus Belcastro, Pahan Gunaratne, Sam Lillis, Katya T. Numbers, Ping-Hsiu Lin, Kathy Nguyen, Minal Tanvir, Simone Reppermund

**Affiliations:** 1 School of Computer, Data and Mathematical Sciences, Western Sydney University, Penrith, NSW, Australia; 2 Translational Health Research Institute, Western Sydney University, Campbelltown, NSW, Australia; 3 Centre for Healthy Brain Ageing, Faculty of Medicine and Health, School of Clinical Medicine, Discipline of Psychiatry and Mental Health, University of New South Wales, Sydney, NSW, Australia

**Keywords:** user interface design, user experience evaluation, activities of daily living, computerised assessments, older adults

## Abstract

The assessment of functional ability in older adults is critical for the early detection and management of age-related cognitive impairment, such as dementia and mild cognitive impairment (MCI). This paper presents a developmental and pilot user experience study of the online implementation and initial user experience evaluation of the Computerised version of the Sydney Test of Activities of daily living in Memory disorders (C-STAM), a new computerised diagnostic screening tool designed to assess functional ability in older adults with and without cognitive impairment. The technical development of the C-STAM involved translating the original STAM tasks into computerised versions, resulting in a scalable, accessible, and user-friendly tool with the potential for integration into regional and remote clinical practices. Key features of the C-STAM include automated scoring, hint options, text-to-speech functionality, a text-magnification tool, and other accessibility enhancements to accommodate diverse physical and cognitive limitations. Within the pilot study of the larger C-STAM trial, secondary feedback was sought from 30 participants (*n* = 13 with and *n* = 17 without cognitive impairment) specifically to assess the C-STAM tool's user experience (UX). This exercise did not evaluate dementia outcomes, which is planned to be reported separately. UX analysis results presented in this paper indicated a positive experience, with an average score of 5.8 ± 1.3 on a seven-point Likert scale, reflecting high perceived usability and user satisfaction. The pilot normal cognition group (*n* = 17) reported higher satisfaction, with an overall score of 6.4 ± 0.4. In comparison, the pilot MCI group (*n* = 6) and pilot dementia group (*n* = 7) reported lower scores of 5.1 ± 1.1 and 4.9 ± 2.1, respectively. Feedback from participants was instrumental in shaping each iteration of the tool and refining the updated version of the C-STAM that will be presented in the main validation study.

## 1 Introduction

The assessment of functional ability in older adults is a critical component in the diagnosis and management of age-related cognitive impairment, particularly dementia and mild cognitive impairment (MCI) (1, 2). These conditions are characterised by a decline in cognitive functions such as memory, attention, and executive function, which can significantly impact an individual's ability to perform basic activities of daily living (ADL) and instrumental activities of daily living (IADL) (3). IADLs represent complex everyday activities such as handling finances, medication management, and shopping, which are essential for independent living and maintaining quality of life (4). Importantly, the main diagnostic criterion that distinguishes a diagnosis of MCI from dementia is the maintenance of functional independence in ADL and IADL (5, 6). Given the increasing prevalence of dementia due to the rising global aging population, there is a substantial burden on healthcare systems worldwide (7). There are limited disease modifying pharmacological interventions for dementia. Therefore, early detection of cognitive decline is crucial for enabling timely interventions that can slow disease progression, alleviate symptoms, and provide support for patients and carers (8, 9). Given the key role that functional ability plays in delineating MCI and dementia, there has been growing research on the development of validated tools to assist with the early detection of cognitive decline based on changes in IADL performance (10).

Traditional methods for assessing IADLs often rely on subjective reports from the person or an informant (e.g., spouse, friend or family member), which can be biased and influenced by various factors such as current mood, personality, and/or lack of insight and may not accurately reflect actual IADL performance (11–13). On the other hand, performance-based measures allow real-time standardised evaluation of IADL performance, where individuals perform a range of IADL tasks under the direct observation of the examiner (14). However, many existing performance-based IADL assessments are too time-consuming or resource-intensive for everyday practice, some requiring administration by specially trained professionals and with insufficient evidence on their psychometric properties (10, 15).

Consequently, the Sydney Test of Activities of daily living in Memory disorders (STAM) was developed and validated as a performance-based instrument to assess everyday activities in a time-efficient and reliable way (15). The STAM requires participants to perform mock versions of nine daily tasks, such as making a phone call and managing medications, that are observed, timed and subsequently scored by a trained researcher. The STAM has demonstrated good psychometric properties, including excellent specificity in distinguishing between normal cognition, MCI and dementia (15), and it has been shown to predict dementia over 4 years (16). Therefore, the STAM serves as a valuable diagnostic screening tool for assessing functional impairment and aiding in the early-stage diagnosis of dementia (15).

Despite the strengths of the STAM as a validated tool to assess IADL, there are a few inherent disadvantages in today's context. The logistical challenges associated with its administration, such as setup time and resource requirements, may impact its efficiency in time-constrained environments. Furthermore, with technological advancements influencing daily activities, many real-life IADLs are now frequently performed on a computer, such as paying bills and shopping, making some tasks included in the STAM (e.g., looking up a number in a phone book) less relevant. In addition to improving efficiency, the transition to digital assessments enhances ecological validity by allowing tasks to be performed in formats that closely mirror contemporary daily activities, such as using digital communication platforms or online financial tools. This digital simulation provides a more authentic representation of functional performance in modern living contexts, particularly for older adults who are increasingly interacting with technology in daily life.

In response to these challenges, a computerised version of the STAM was envisioned to realistically simulate relevant IADLs and accurately assess functional performance in older people with or without cognitive impairment. The development of a scalable computerised assessment for IADLs, which builds upon the foundation of the validated STAM, allows for a streamlined and standardised functional assessment. A computerised assessment has several advantages over traditional performance-based measures, including a shorter assessment time that facilitates administration in time-restricted settings and greater cost-efficiency due to automated administration and scoring, eliminating the need for additional resources (e.g., props) or training (17). A computerised version of the STAM supports remote assessments, which have become increasingly important due to the COVID-19 pandemic, enabling clinicians to reach individuals in remote and rural areas who may lack access to specialists and face long waiting and travel times (18). Moreover, the computerised assessment provides greater measurement precision and automated data exporting and reporting, thereby enhancing accessibility, efficiency, and reliability in both clinical and research settings (17).

This work presents the online implementation efforts and initial user experience evaluation of the Computerised version of the Sydney Test of Activities of daily living in Memory disorders (C-STAM). We hypothesise that the C-STAM would demonstrate acceptable user experience (UX) across participant groups, providing preliminary evidence to support its future use in clinical and research settings. Thus, the main objective of this project was to translate, yet accurately represent, the activities and functional domains captured from the original STAM into online (computerised) versions, with the goal of developing a scalable and accessible tool to assess functional ability that is feasible and acceptable to target-end users (i.e., older adults with and without cognitive impairment) that has the potential for future integration into clinical practices.

## 2 Materials and methods

### 2.1 Development and design process

The online implementation of the nine new activities for the C-STAM was conducted with the active involvement of all stakeholders, including clinicians and subject matter experts, lived experience consumers and carers, and clinical research and Information Technology (IT) teams. Initially, all tasks of the original STAM were reconceptualised for the C-STAM prototype using Microsoft PowerPoint slides, created by the clinical research team to communicate their ideas to subject matter experts. These prototypes were assessed by an international panel of experts in IADL, including researchers, occupational therapists, neuropsychologists, and IT specialists for feasibility, acceptability, and validity via a modified Delphi survey. The findings of the Delphi study are planned to be presented in a separate publication. The final nine items from the Delphi survey were then presented to an advisory group consisting of individuals with and without cognitive decline, as well as carers, to assess their difficulty and feasibility. Throughout this process, the IT design team worked closely with the clinical research team to explore how the original STAM activities could be effectively implemented as computerised activities and what feedback from the consumer advisory group could be effectively implemented. During this period, the IT team also began technical development and infrastructure setup for the C-STAM. This involved organising development and production server environments and selecting the appropriate software and development tools.

After finalising the design elements of the user interface for each item for the C-STAM based on feedback from the Delphi panel and advisory group, the computerised version of all assessment items was implemented. The implementation process involved several testing iterations to refine the C-STAM activities. Feedback loops from various user groups informed each iteration. The C-STAM was first tested for feasibility and user experience (UX) in a pilot study with participants with (*n* = 13) and without (*n* = 17) cognitive impairment. There were three groups of participants in the pilot study: (i) pilot dementia group; (ii) pilot mild cognitive impairment (MCI) group, and (iii) pilot normal cognition group. Purposive sampling was utilised to recruit older adults for the pilot and main validation study via self-selection, whereby interested individuals responded to flyers and advertisements by contacting the research team via email or phone. Eligible participants were at least 60 years old, could communicate in English without an interpreter, could use a computer mouse and keyboard (basic skills), lived in Sydney, and had someone who knew them well and was in contact with them at least once a week. Pilot participants' (*n* = 30) feedback and user experience questionnaire responses were used to develop a final version for a full validation study to test the psychometric properties of the C-STAM.

### 2.2 Choice of technology framework

This project utilised two primary technological frameworks: Unity game development platform (19) for the front end and PHP (Hypertext Processor) Laravel web development framework for the back end (20). Unity is a cross-platform game engine widely used to develop interactive content. It supports a broad range of browsers and devices, making it an ideal choice for front-end development due to its graphical capabilities and flexibility. Despite certain limitations associated with using Unity as a game development environment, including runtime overhead, it was selected for its flexibility and suitability for creating interactive, graphics-intensive front-end applications. PHP Laravel is a web application framework that supports accelerated development, scalability options and security, making it highly suitable for backend research data management. A limitation of this framework is that newer technologies may be available, however, it was chosen for its cost-effectiveness, implementation suitability, and overall appropriateness for the project. While other technologies, such as React for the front end and Node.js for the back end, could have been considered, Unity was chosen for its real-time interaction capabilities, and Laravel was selected for its comprehensive ecosystem and security features, which were suitable for efficient data handling and scalability in the C-STAM project.

In addition to the primary technologies, several artificial intelligence (AI) tools were integrated into the project to enhance audio and visual elements. For text-to-speech conversion, Amazon Web Services (AWS) Polly (21) and ElevenLabs (22) were utilised, providing clear and lifelike audio output. For image generation, DALL-E (23) was employed, enabling the creation of unique, non-copyrighted visual content. The use of these technologies significantly accelerated the development process, allowing for high-quality, cost-effective visuals and audio.

### 2.3 User experience evaluation

For the pilot study, participants completed the C-STAM as well as a brief cognitive screening test (24), an abbreviated version of an Occupational Therapy (OT) test of daily function (25), and questions about mood and recent health changes. Prior to the in-person assessment, pilot participants also completed a battery of questionnaires about their health, medical history, social and other lifestyle factors, and levels of physical activity.

When completing the C-STAM, participants’ responses, keyboard strokes, mouse clicks, hints accessed, and time taken to complete each task were all recorded. Subsequently, users were asked to complete a user experience assessment questionnaire for the C-STAM tool. The questionnaire was adapted and modified from the IBM Computer Usability Satisfaction Questionnaire (CUSQ) (26) and the Post-Study System Usability Questionnaire (PSSUQ) (27), which are widely used questionnaires for assessing user satisfaction with computer systems (28). The questionnaire consisted of 18 items, each rated on a seven-point Likert scale ranging from 1 (strongly disagree) to 7 (strongly agree). Higher scores indicate greater degrees of perceived usability or satisfaction with the C-STAM tool. The scores for the 18 items were used to calculate the overall score, as well as three subscales, as follows:

- Overall: The average scores of questions 1 to 18
- System Usefulness (SYSUSE): The average scores of questions 1 to 7
- Information Quality (INFOQUAL): The average scores of questions 8 to 13
- Interface Quality (INTERQUAL): The average scores of questions 14 to 17

The overall and subscale scores were compared to identify areas that needed improvement. In addition to the 18 questionnaire items, users were asked to respond to two additional open-ended questions to list the negative aspects and the most positive aspects of the C-STAM tool. One-way ANOVAs were used to examine mean differences between groups for continuous variables, with results presented as mean ± standard deviation (SD). For variables showing significant differences in the ANOVA, Tukey HSD *post-hoc* tests were conducted to identify pairwise group differences. Categorical variables were summarised using frequencies and percentages. All analyses were performed using the latest version of R Studio (v2025.09.2 + 412), with a significance level set at *p* < 0.05. The responses to the open-ended questions were analysed using an inductive thematic approach, where two researchers independently coded the data to identify patterns and emerging themes. The coding was then compared and refined through discussion to reach consensus. The sample included a mix of older adults with normal cognition, mild cognitive impairment, and dementia, providing sufficient diversity to explore key themes related to usability, accessibility, and user experience. At the time of this writing, the main validation study is in progress; thus, user experiences concerning the main study participants are not reported here.

## 3 Development and design of the C-STAM

### 3.1 Development of the C-STAM from STAM

The development of the C-STAM marked a significant transition from the performance-based, in-person STAM that was observed and scored by an assessor to a self-completed computerised tool that is autonomously administered and scored. This process involved reimagining the original STAM assessment tasks within a digital framework, ensuring that the core functional domains were preserved while utilising the advantages of computer-administered assessments, such as standardisation, precision, and the ability to collect and score detailed response data automatically.

The original STAM consists of nine tasks assessing the following domains of function: communication (looking up and making a phone call), dressing (putting on a shirt), handling finances (paying a bill by check), managing everyday activities (preparing the check for mailing), orientation (time orientation—reading the time and setting the alarm), medication management (managing medications using a dispenser), shopping (choosing items to make a simple recipe), counting money (calculating cost and counting money), and memory (recalling activities completed) (15).

During the transition from the STAM to the C-STAM, the tasks and scenarios from the original STAM were adapted for a digital environment, ensuring they remained relevant and realistic in a computerised format. This involved redesigning tasks to be interactive and engaging while retaining their core objectives. For example, a task that originally involved physically sorting medication was transformed into a virtual task in which users dragged and dropped medication icons into the correct compartments of a digital pillbox.

The C-STAM consists of two practice tasks and nine assessment tasks, outlined as follows:

- Practice Task 1—Making a beverage (domain: managing everyday activities)
- Practice Task 2—Setting a timer (domain: orientation)
- Task 1—Look up GP's (i.e., general practitioner) phone number (domain: communication)
- Task 2—Call GP to refill script (domain: communication)
- Task 3—BPay (i.e., bill payment) for electricity bill (domain: handling finances)
- Task 4—Online grocery shopping (domain: shopping)
- Task 5—Calculating shopping total after “special” items added (domain: counting money)
- Task 6—Dressing (domain: dressing)
- Task 7—Navigating directions (domain: managing everyday activities)
- Task 8—Managing medications (domain: medication management)
- Task 9—Memory task (domain: memory)

The scoring of the C-STAM is similar to that of the original STAM (15). Each task is scored on a 4-point scale, and points are awarded for completing the various components of each task correctly, with a point awarded for not using any hint. For each task, users receive 1 point for each correct component (maximum 4 points per item) and 0 points for an incorrect component or using a hint/s. The total score is calculated as the sum of scores for all nine tasks, with total scores ranging from 0 to 36, and higher scores indicating better performance.

### 3.2 Technical development and user interface design

The online implementation and user interface design of the C-STAM integrated various technologies and development principles, resulting in a comprehensive and user-friendly assessment tool. The C-STAM was developed using the Unity application, a versatile platform commonly employed in video games and interactive software development (19). The C-STAM was deployed as a web-based application using WebGL, enabling it to run on modern web browsers across various operating systems and platforms. The C-STAM assessment tool guides users through a series of interactive tasks, provides scoring based on predefined criteria, offers assistance through hints, and accommodates users with various accessibility needs (e.g., text-to-speech and text-magnifying options).

The architecture of the C-STAM comprises two main components: a front-end Unity application and a back-end PHP Laravel server. The front-end component is the interactive interface through which users engage with a series of tasks designed to assess their functional abilities. The Unity application encompasses the bulk of the assessment's logic, including scene/task traversal, scoring mechanisms, and providing hints and narration to guide users through the assessment. On the other hand, the back-end PHP Laravel server is responsible for storing the data from the assessment and other pertinent tracking information. In line with the principle of Separation of Concern (SOC), the server is primarily focused on managing data operations (Create, Read, Update and Delete—CRUD) and does not handle any of the assessment logic (29). This clear delineation of responsibilities ensures that the Unity application can concentrate on the assessment's functionality while the Laravel server efficiently handles data management. This design choice also reduces the computational load on the server, enabling it to hold a larger capacity of incoming requests with minimal performance impact (30).

A key aspect of the user interface design is the C-STAM scoring system, which was implemented through both active and passive methods. Active scoring assigns a score when a specific event is triggered, such as clicking a button. In contrast, passive scoring calculates a score when the user moves to the next task. The design of the scoring system was focused on simplicity and modularity, with each scoring criterion linked to a specific function, making the scoring logic easy to debug, unit test, and modify. This design philosophy also prevented the embedding of scoring logic deep within other functions in the code, thereby reducing the likelihood of errors and making the system more maintainable.

Given how significantly the use of hints impacts participants' scores on the C-STAM, special consideration was provided to develop the hint features. When a user clicks the hints button, they are first asked for confirmation: “You are about to use the hints for this task. Are you sure you want to continue?” The scoring criteria were developed in such a way that if a user utilises any hints for a task, they lose a point for that particular task but are not penalised multiple times if multiple hints are used for the same task. Additionally, a point for not using hints is only awarded if the attempt has begun, which was a feature designed to ensure fairness in scoring. Conversely, if a user clicks the “Skip” button, they are prompted for confirmation to skip the task, along with an indication that they will not be able to go back. However, they still receive points depending on how far they have progressed in the task.

To accurately score the C-STAM, it was essential to track additional variables that could impact participants’ scores. These variables include whether a task was skipped, if and how many of each type of hint were used, if the task attempt was begun, or whether the zoom-in or Text-to-Speech (TTS) functions were utilised. Key factors, such as the time taken to complete each task, the use of hints, and the total score, are incorporated into the scoring models, enabling adjustments to the scores as needed, making it vital to capture this information. Additionally, this detailed tracking provides a comprehensive understanding of user interactions, along with informing us about different users' needs and behaviours. Furthermore, different tasks within the assessment have specific scoring logic. For instance, in the “Call GP to refill script” task, if a user calls an incorrect number and then the correct number, they still lose a point. Conversely, in the “Online grocery shopping” task, if a user picks the wrong item but corrects it, they still receive the point. This distinction ensures that mistakes in certain tasks, where actions are reversible, do not unfairly penalise the user, whereas irreversible mistakes in other tasks do impact the score.

Another significant aspect of the user interface design is the event collection and replaying feature, which was deemed essential for debugging issues in production environments and providing concrete evidence for bug reports. Moreover, this feature enabled the research team to review user experiences and, in some instances, help the team resolve ambiguities in participant scores when needed. The Unity app, which forms the core of the C-STAM, enabled the recording of user interactions, such as mouse clicks, keyboard inputs, and scroll actions. This data is then stored and uploaded in a single web request alongside the scoring for each assessment task, which could then be used to recreate the entire user experience, providing valuable insights into user behaviour and tool performance.

String tables provided by Unity were implemented to further streamline the user interface design of the C-STAM (31). String tables are a data structure that allows the IT team to conveniently store and access lists of strings, along with enabling easy text modification based on feedback from users and advisory groups. The localisation tables in Unity were utilised to implement narration within the C-STAM assessment, which streamlined the process of displaying text and integrating text-to-speech functionality. In the early stages of the project, any adjustments to the wording of prompts required involvement from the IT team for modification and inclusion in the subsequent releases. Therefore, an operational pipeline was developed for narration text to ensure that any necessary changes to the text could be made seamlessly after development. This pipeline enabled the research team to modify an online spreadsheet, which was accessed by the IT team while building a new release, and the changes were subsequently implemented in the Unity application, and the text-to-speech assets were updated programmatically.

### 3.3 Overview of the C-STAM user interface

**Figure 1:**
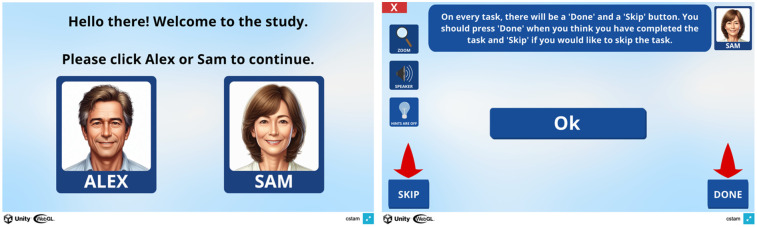
The user interface of the C-STAM along with accessibility features. Screenshot(s) from C-STAM application, UNSW Sydney (https://www.unsw.edu.au/research/c-stam).

As shown in Figure 1, users are first provided with the option of selecting between two avatars (i.e., Alex and Sam). Users are then prompted to enter full-screen mode by pressing the blue button on the bottom-right of the screen. The user interface incorporates several accessibility features, including a zoom button, a speaker button, and a hints button. Positioned on the left side of the interface is a zoom or magnifying glass icon, and activating this feature provides a magnifier for enlarging small text. The speaker icon is the Text-to-Speech button, which speaks out the on-screen text for those with vision issues or who prefer to listen instead of reading. The hints button is the lightbulb icon, which is available for users if they are stuck on a task and need some advice on what to do next. However, users are encouraged to attempt tasks without using hints. Moreover, each task within the interface includes both a “Done” and a “Skip” button. Users are advised to click “Done” when they believe they have completed a task and “Skip” when they would like to skip the task. The red cross button on the top left is the exit button, which users are advised to press only if they want to abandon all progress and quit the C-STAM. An iterative user-centred design process was employed for the design of the C-STAM user interface, informed by continuous feedback from pilot participants, advisory board members, and subject matter experts at multiple development stages of the C-STAM.

### 3.4 Design considerations for the C-STAM

Several critical considerations were essential in designing the C-STAM to ensure its effectiveness and accessibility for a diverse range of users, particularly older adults with cognitive impairment. Key considerations included integrating accessibility features to cater to users with various impairments, such as vision or hearing challenges or difficulties with thinking. These accessibility features were primarily based on the consumer advisory group and pilot participants' feedback.

One of the key accessibility features integrated into the C-STAM is the Text-to-Speech (TTS) functionality, which is particularly important for users with vision impairments or reading difficulties who rely on audio narration (32). AWS Polly's neural-voice TTS service employs a neural network to enhance the quality of the produced speech, which provides a more natural and comforting voice compared to traditional TTS solutions, thereby putting users at ease during the assessment (21, 33). Moreover, the use of voice synthesis AI models to replicate the voice of a voice actor and enabling the conversion of arbitrary text into realistic spoken words was essential for providing a more authentic auditory experience for users. ElevenLabs is an AI voice generator known for its ability to produce highly realistic and customisable speech from text inputs (22). The use of ElevenLabs allowed us to generate a native Australian speaker's voice, which was aimed at further enhancing comfort, relatability and the overall user experience (22). The TTS feature in the C-STAM is used specifically for narration, rather than reading all arbitrary text on the screen as with a typical screen reader. This narration covers approximately 80% of the text within the assessment, representing a significant step in enhancing the accessibility of the C-STAM assessment tool. The TTS functionality was especially important in tasks with lengthy instructions such as the “Look up GP's phone number” task, along with tasks with auditory component such as “Call GP to refill script” where users dial the number, listen carefully to the options, and select the correct option to renew their prescription and select the correct GP.

A magnification tool was integrated into the Unity application to cater to the needs of users with vision impairment (34). This tool enables users to enlarge text and other elements within the assessment, making it easier to read and engage with the content. The implementation of the magnification tool involved creating a new canvas with a User Interface (UI) image and camera, adjusting the camera settings for zoom, and ensuring that this magnified view tracks the cursor. By embedding the magnification feature into every scene as required, the tool becomes more accessible for individuals with vision challenges.

**Figure 2:**
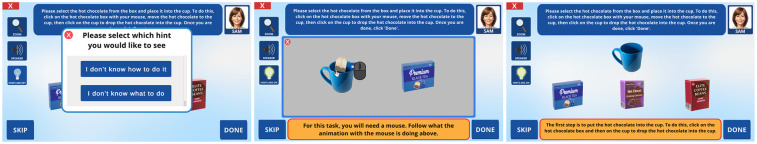
The hints feature of the C-STAM along with the variation of hints provided. Screenshot(s) from C-STAM application, UNSW Sydney (https://www.unsw.edu.au/research/c-stam).

Another essential design consideration was the hints feature, which was aimed at guiding the users through the C-STAM assessment. When users seek to use the hints for a particular task, they are first asked to select which type of hints they want to see. As shown in Figure 2, two options are presented: “I don’t know how to do it” and “I don’t know what to do”. The first type of hint seeks to provide technical assistance to help the user understand which buttons to press, catering to those who may have less computer literacy and/or confidence. If a user selects this type of hint, they are shown a brief animated tutorial demonstrating how to click, drag and drop, or type using visual representations of a computer, mouse, and keyboard icons. The second type of hint seeks to assist users who may have forgotten what they were supposed to do or need clarification on the task's objective and was designed to assist users with memory difficulties. These two distinct types of hints were designed to allow users to progress when stuck within a task without needing intervention from the interviewer or clinician.

Key design considerations in the development of the C-STAM also included aspects such as contrast, colour, vibrancy, and realism. The design focused on enhancing contrast and avoiding problematic colour combinations, such as light shades of blue and white with minimal contrast, which can be challenging for some older users to differentiate. An essential design principle was ensuring that every interface element had a background, thus making designs independent of their backdrop and avoiding colour contrast issues. Monotone colours such as black and white were also avoided to ensure the assets appeared as realistic as possible, mimicking the challenges in everyday life accurately. Moreover, based on published research, colour vibrancy was used to create a sense of liveliness to help users feel comfortable, confident, and engaged in the assessment (35).

**Figure 3:**
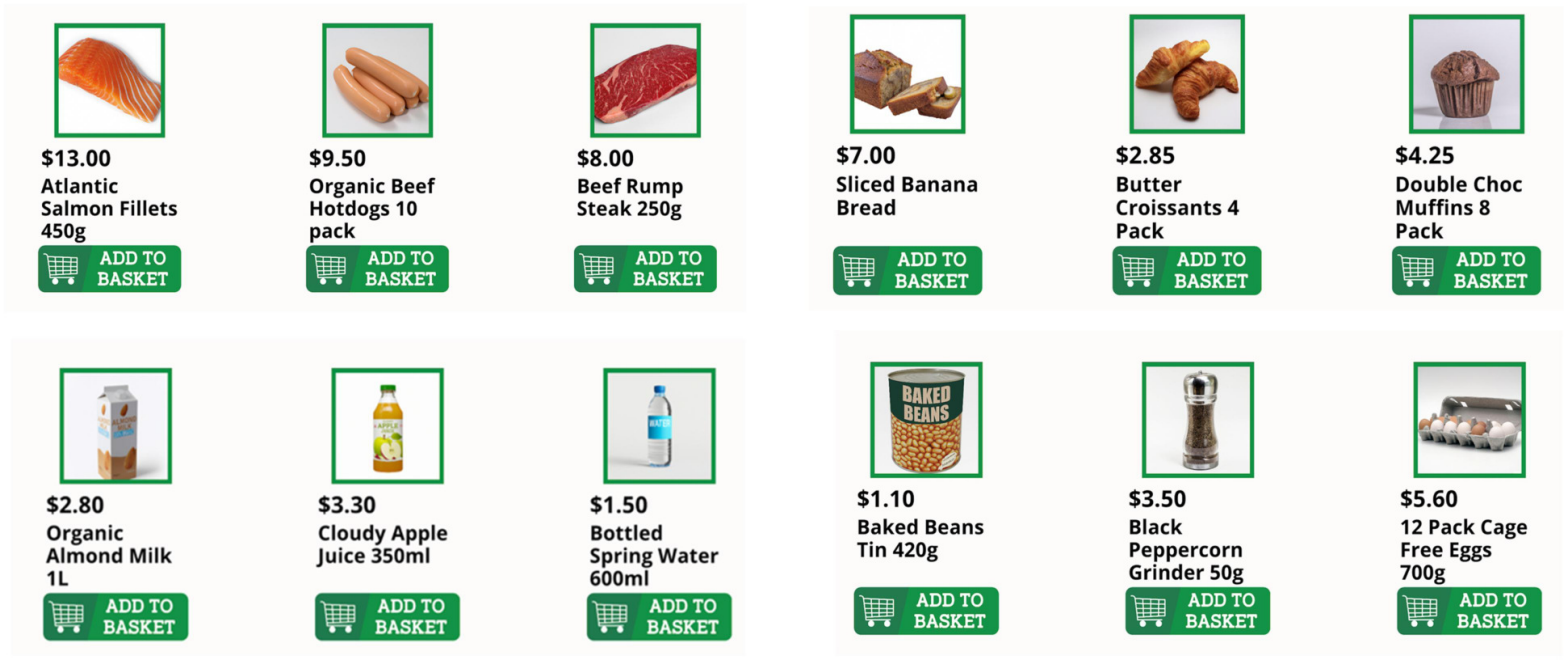
Example images of realistic grocery items generated with DALL-E (https://openai.com/index/dall-e-2/) and used in the “Online Grocery Shopping” task in C-STAM.

The use of AI-generated imagery, particularly through the DALL-E model, allowed for the efficient creation of assets for the C-STAM (23, 36). The AI-generated images were especially effective for depicting everyday objects, requiring minimal post-processing. DALL-E was particularly useful for tasks that required non-copyrighted content, such as the “Online grocery shopping” task where users were provided with images of various grocery items (e.g., Roma tomatoes, deli sliced boneless ham, sliced peaches tin, pressed orange juice) to be added to the shopping cart as part of the assessment (Figure 3). However, this approach was less effective in generating human faces and features, such as the avatar profile images, highlighting a limitation in the current capabilities of AI-generated imagery.

Another essential design consideration was perpetual context, especially for users with cognitive impairment such as short-term memory loss. The C-STAM interface was designed to be familiar, consistent, and obvious in every scene. Clear sections were defined, and text was added to interface buttons to facilitate understanding and navigation. Avoiding the use of images or icons alone for buttons further reduced ambiguity, particularly for users with lower technical literacy (37).

### 3.5 Evolution of user interface designs of the C-STAM

**Figure 4:**
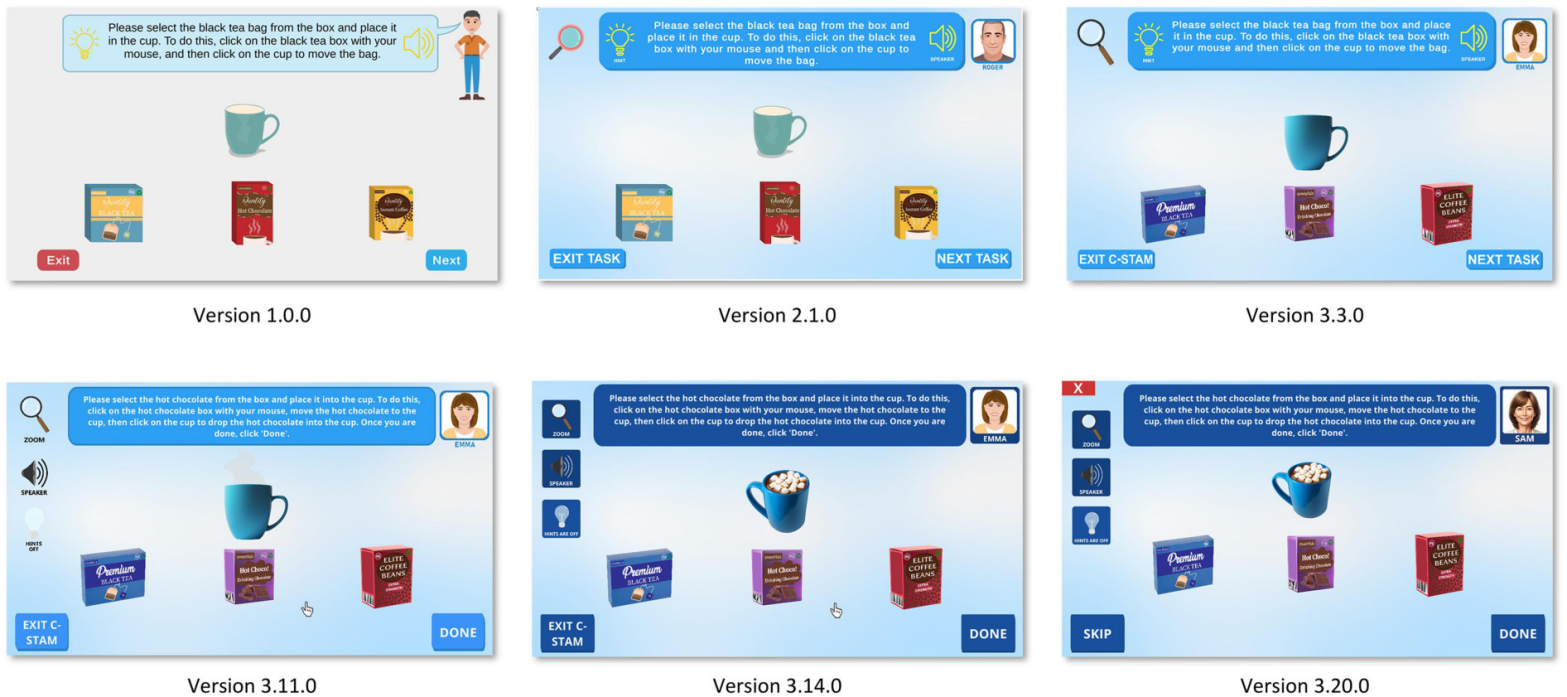
Evolution of user interface of the C-STAM. Screenshot(s) from C-STAM application, UNSW Sydney (https://www.unsw.edu.au/research/c-stam).

**Figure 5:**
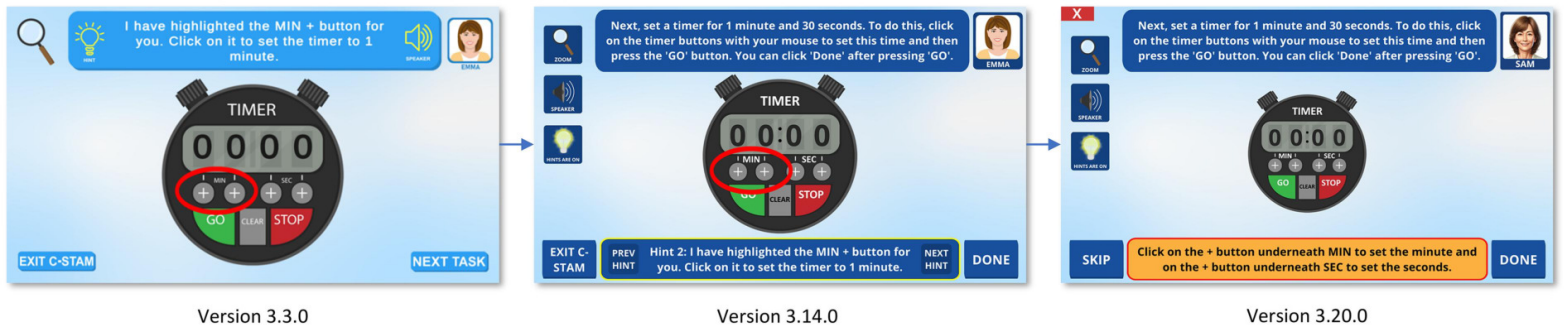
Evolution of hints in the user interface of the C-STAM. Screenshot(s) from C-STAM application, UNSW Sydney (https://www.unsw.edu.au/research/c-stam).

Figures 4, 5 showcase the major evolutions in the C-STAM interface as per feedback from consumers, advisory board members, and subject matter experts from the research team, along with the participants with and without cognitive impairment from the pilot study. Initially, all tasks embedded in the C-STAM were conceptualised in Microsoft PowerPoint and subsequently implemented as a Unity application. Figure 4 illustrates the evolution of the user interface of the C-STAM, taking “Practice Task 1—Making a beverage” as a reference. Significant changes are evident from Version 1.0.0 to 2.1.0, including alterations in the colours within the interface, integration of the magnification tool, and the inclusion of the avatar profile image (i.e., Roger). Version 3.3.0 showcases further refinement of images (e.g., cup and beverage boxes) and icons (e.g., magnification tool). In Version 3.11.0, the hints and speaker buttons were relocated from the task description panel to the left of the interface (below the magnification tool) to consolidate accessibility features. Notable changes from Version 3.11.0 to 3.14.0 include adjustments to the colour profile for enhanced contrast (i.e., light blue to dark blue) and modifications to images for a more realistic appearance (e.g., marshmallows within the cup). Finally, essential modifications in Version 3.20.0 include the incorporation of a more realistic profile image of the avatar (without appearing ageist), and the change of avatar names to include gender-neutral names (i.e., Roger and Emma changed to Alex and Sam). Figure 5 further demonstrates the evolution of the hints feature in the user interface, using “Practice Task 2—Setting a timer” as a reference, with hints replacing the task description in Version 3.3.0, the hints panel appearing at the bottom of the screen in Version 3.14.0, and the hints panel being further highlighted for clarity in Version 3.20.0. Some challenges were encountered during development, including ensuring accessibility for users with visual or cognitive impairments, creating realistic AI-generated imagery for avatars and objects, and designing fair scoring logic for tasks with reversible and irreversible actions. These were addressed through iterative testing, integration of TTS and magnification features, consultation with consumer advisory groups, and continuous refinement of scoring criteria to ensure usability.

## 4 User experience evaluation of the C-STAM

### 4.1 Quantitative results of user experience evaluation

A total of 30 participants completed the user experience assessment questionnaire as part of the feasibility testing of the C-STAM tool in a pilot study involving individuals with and without cognitive impairment. Of the 30 participants, 23.3% (*n* = 7) were in the pilot dementia group, 20.0% (*n* = 6) were in the pilot mild cognitive impairment (MCI) group, and 56.7% (*n* = 17) were in the pilot normal cognition group. As shown in Table 1, the average age of participants was 71.7 ± 5.7 years, and 50% (*n* = 15) were female. The majority of participants identified as Caucasian (90%; *n* = 27) and had a tertiary education (80%; *n* = 24).

**Table 1:** Characteristics of study participants (*n* = 30).

Characteristics	All participants (*n* = 30)	Pilot dementia group (*n* = 7)	Pilot mild cognitive impairment group (*n* = 6)	Pilot normal cognition group (*n* = 17)	*p*-value^a^
Age (in years)	71.7 ± 5.7	74.1 ± 4.1	73.7 ± 3.2	70.0 ± 6.4	0.170
Sex					0.043
Male	15 (50%)	5 (71.4%)	5 (83.3%)	5 (29.4%)	
Female	15 (50%)	2 (28.6%)	1 (16.6%)	12 (70.6%)	
Caucasian ethnicity	27 (90%)	7 (100%)	5 (83.3%)	15 (88.2%)	0.765
Completed tertiary education	24 (80%)	6 (85.7%)	5 (83.3%)	13 (76.5%)	0.999

a Group comparison was conducted using one-way ANOVA for age, and Fisher's exact for sex, ethnicity and education.

**Figure 6:**
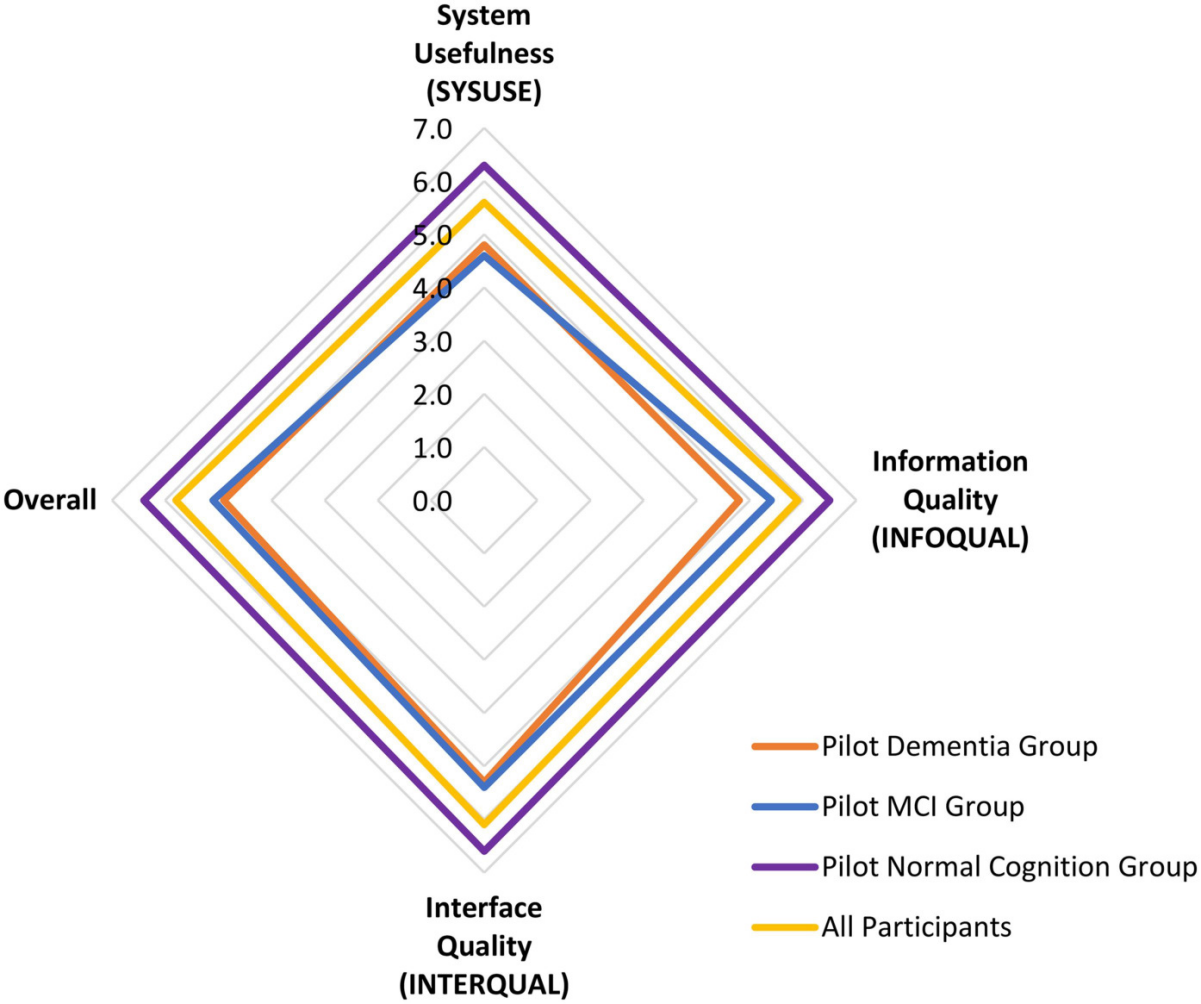
Summary of results from the user experience assessment questionnaire for the C-STAM assessment.

The summary of results from the user experience assessment questionnaire is presented in Figure 6. When considering all 30 participants across groups, the overall average scores for System Usefulness (SYSUSE), Information Quality (INFOQUAL), and Interface Quality (INTERQUAL) were 5.6 ± 1.5, 5.9 ± 1.3, and 6.1 ± 1.3, respectively, resulting in an overall score of 5.8 ± 1.3. Higher scores across all categories were reported by the pilot normal cognition group (SYSUSE: 6.3 ± 0.6; INFOQUAL: 6.5 ± 0.4; INTERQUAL: 6.6 ± 0.5), with an overall score of 6.4 ± 0.4. In comparison, the pilot MCI group had a lower overall score (5.1 ± 1.1) and across categories (SYSUSE: 4.6 ± 1.4; INFOQUAL: 5.4 ± 1.3; INTERQUAL: 5.4 ± 0.8). The pilot dementia group had the lowest overall score (4.9 ± 2.1), as well as across most categories (SYSUSE: 4.8 ± 2.3; INFOQUAL: 4.8 ± 2.2; INTERQUAL: 5.3 ± 2.4).

One-way ANOVA indicated significant differences between groups for all categories (SYSUSE: *p* = 0.013; INFOQUAL: *p* = 0.009; INTERQUAL: *p* = 0.031; overall: *p* = 0.008). *post-hoc* Tukey HSD tests showed that differences were most pronounced between the Pilot Dementia Group and Pilot Normal Cognition Group, particularly for Information Quality (*p* = 0.011) and overall score (*p* = 0.015) as shown in Table 2.

**Table 2:** User experience scores across pilot groups and *post-hoc* comparisons.

	System usefulness (SYSUSE)	Information quality (INFOQUAL)	Interface quality (INTERQUAL)	Overall
All Participants	5.6 ± 1.5	5.9 ± 1.3	6.1 ± 1.3	5.8 ± 1.3
Pilot Dementia Group	4.8 ± 2.3	4.8 ± 2.2	5.3 ± 2.4	4.9 ± 2.1
Pilot MCI Group	4.6 ± 1.4	5.4 ± 1.3	5.4 ± 0.8	5.1 ± 1.1
Pilot Normal Cognition Group	6.3 ± 0.6	6.5 ± 0.4	6.6 ± 0.5	6.4 ± 0.4
*p*-value^a^	0.013	0.009	0.031	0.008
Post-hoc Tests (Tukey HSD)^b^				
Group 1 vs. Group 2	0.961	0.586	0.960	0.918
Group 1 vs. Group 3	0.053	0.011	0.061	0.015
Group 2 vs. Group 3	0.036	0.151	0.117	0.062

a One-way ANOVAs were used to test for mean differences between groups.

b Tukey HSD *post-hoc* tests were conducted for significant findings; group 1 was the pilot dementia group, group 2 was the pilot MCI group, and group 3 was the pilot normal cognition group.

**Figure 7:**
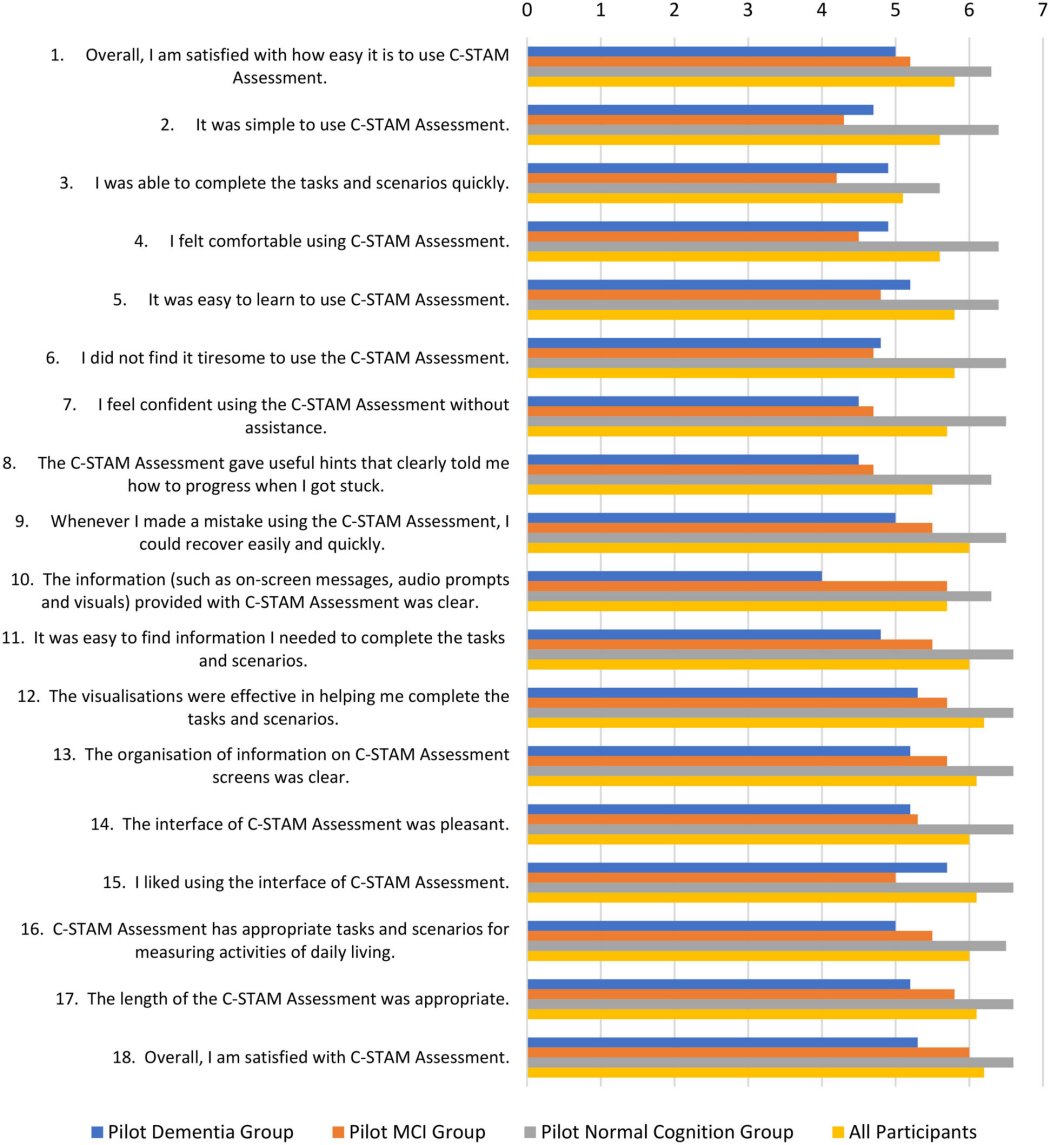
Participants’ responses on the 18 items of the user experience assessment questionnaire for the C-STAM assessment.

Figure 7 illustrates the variations in participants' responses to the 18 items of the user experience assessment questionnaire for the C-STAM, stratified by group, highlighting differences in feedback on the system's functionality and user experience across participant categories.

### 4.2 Qualitative results of user experience evaluation

Participant feedback on the C-STAM was collected as free-text responses and analysed thematically. Based on the responses of the 30 participants regarding the positive and negative aspects of the C-STAM tool, two key themes were identified: user interface and usability, and accessibility and adaptability. Direct quotations from the participants are presented to illustrate the identified themes and labelled as per the participant type and ID (e.g., *Pilot MCI group, Participant 1*)*.*

#### 4.2.1 User interface and usability

Many pilot participants (*n* = 14) commented on the simplicity and clarity of the C-STAM tool, which made it easier to use and understand.

“Very simple interface” (Pilot normal cognition group, Participant 13)

“It was clear and easy to navigate” (Pilot normal cognition group, Participant 16)

“Quite intuitive .… easy to understand” (Pilot MCI group, Participant 6)

“Simple language” (Pilot dementia group, Participant 4)

A few participants (*n* = 6) indicated that the features of the C-STAM tool, such as hints and audio, were pivotal for having a positive experience with the user interface and usability.

“The most positive aspect of the C-STAM tool was … hints” (Pilot dementia group, Participant 5)

“It was great having the audio. The done button was consistent on each page. The font and size of the writing was big enough to read without having to adjust” (Pilot normal cognition group, Participant 17)

However, some participants (*n* = 5) indicated having difficulties with unclear instructions and challenges in navigating or understanding certain aspects of the system.

“Some instructions were not completely specific, especially finding Dr Gary's phone number” (Pilot normal cognition group, Participant 5)

“Had to read instructions a few times to understand how to do it. But that is probably my comprehension skills” (Pilot normal cognition group, Participant 11)

“Early instructions were a bit vague. Maybe a bit of a pre-primer [introduction] at the beginning, e.g., There are multiple components to answer these. Take your time to understand all components of a task” (Pilot dementia group, Participant 3)

A few participants (*n* = 5) also found the design of the C-STAM to be challenging, especially those who were less familiar with computers or specific tasks within the C-STAM.

“Harder at first if not used to using mechanics of computer” (Pilot MCI group, Participant 2)

“Those not familiar with online shopping and computers would find it difficult. So, I guess a trial would help to clarify the questions for users” (Pilot normal cognition group, Participant 14)

#### 4.2.2 Accessibility and adaptability

Most participants (*n* = 19) commented that they found the C-STAM tool to be easy to follow and use and appreciated its accessible design. They noted that once started, the system was straightforward, making it accessible for both users and assessors.

“Easy to follow once started” (Pilot MCI group, Participant 2)

“Easy for assessor to use” (Pilot normal cognition group, Participant 3)

“Was easy to follow instructions and clear” (Pilot dementia group, Participant 7)

A few participants (*n* = 4) also expressed enthusiasm about the potential of the C-STAM as a more engaging and reliable diagnostic screening tool compared to traditional tasks for cognitive assessments, with suggestions provided on enhancing the effectiveness of the C-STAM tool.

“Overall, I enjoyed the experience and hope that a reliable testing tool for assessors will be an outcome. It will be a bit more interesting than the current pen and paper tasks”. (Pilot normal cognition group, Participant 1)

“I liked this tool and could see how it would be good for cognitive assessments … if this test is to be repeated annually, please change the scenarios so they are not the same”. (Pilot dementia group, Participant 2)

However, some participants (*n* = 6) expressed concerns about the accessibility of the C-STAM and its adaptability to different user needs. Some of the issues reported were difficulties with mouse and keyboard functionality and lengthy assessment time.

“I was somewhat startled that my finger presses on the pad and mouse did not work as I am used to on my Apple laptop pad” (Pilot normal cognition group, Participant 1)

“Unfamiliar keyboard … strange logic” (Pilot dementia group, Participant 4)

“Quite long assessment especially for a person with dementia” (Pilot normal cognition group, Participant 3)

### 4.3 Discussion of findings of user experience evaluation

The user experience evaluation of the C-STAM tool provided valuable insights into its usability, functionality, and overall user satisfaction as a diagnostic screening tool for assessing functional ability in older adults with and without cognitive impairment. The findings revealed both the strengths and limitations of the C-STAM tool from the perspectives of diverse user groups, including individuals with normal cognition, MCI, and dementia. It is important to note that cultural and linguistic factors may influence user interactions, particularly for users from non-English speaking backgrounds, which could affect accessibility and the generalisability of the findings. It is essential to note that participants may have interacted with different versions of the C-STAM tool during the study, and some limitations identified may have already been addressed in subsequent iterations of the tool. Feedback from participants in this pilot study was crucial in shaping each iteration of the tool and played a vital role in refining the updated version of the C-STAM that is currently being tested in the main validation study.

This pilot study demonstrated a generally positive user experience with the C-STAM tool, with an overall average score of 5.8 ± 1.3 on the seven-point Likert scale, reflecting a high level of perceived usability and user satisfaction. This finding aligns with existing literature that emphasises the importance of co-design principles to ensure accessibility and engagement, particularly for digital tools targeting older adults (38, 39). When stratified by group, the pilot normal cognition group reported the highest levels of satisfaction (6.4 ± 0.4), followed by the pilot MCI group (5.1 ± 1.1) and pilot dementia group (4.9 ± 2.1). The greater variability observed in the pilot dementia group may be attributed to the inclusion of individuals at varying stages of dementia, whose differing cognitive and functional abilities likely contributed to a wide range of user experiences with the tool. Given the exploratory and descriptive nature of this pilot study, these differences should be interpreted cautiously and can only be taken as preliminary evidence of the tool's ability to differentiate cognitive status. Moreover, given that individuals with cognitive impairment often encounter barriers when engaging with complex digital tasks, the relatively lower scores and feedback from the MCI and dementia groups led to the recognition of areas for further refinement (e.g., hints showing how to complete task components), particularly for users with cognitive impairment (40). While the validation study is still underway, the differences in user experiences among the three groups provide a pre-emptive indication that the C-STAM tool may differentiate between individuals with and without cognitive impairment. This also emphasises the need for additional supports such as stepwise guidance and enhanced hints in future versions. Nonetheless, these findings provide preliminary insights into how the tool might be tailored to support different cognitive profiles.

The findings from the three subscales of the user experience evaluation provided a detailed understanding of the positive features and areas for improvement of the C-STAM tool. The System Usefulness (SYSUSE) subscale revealed high perceived practicality and effectiveness (average score: 5.6 ± 1.5), with most participants finding the tool effective and easy to use. However, some noted that the assessment could feel lengthy and tiresome, particularly for individuals with cognitive impairment. This feedback underscores the importance of considering assessment length as a factor that may impact test burden and cognitive load for older adults (41). The Information Quality (INFOQUAL) subscale received positive feedback on the clarity and relevance of the information provided (average score: 5.9 ± 1.3). However, some participants expressed difficulty understanding certain tasks (e.g., the navigating directions task). These reported challenges led to refinements in subsequent iterations of the tool, with a focus on providing clear and straightforward information while designing digital tools for older adults (42). The Interface Quality (INTERQUAL) subscale received high ratings for the design and layout of the C-STAM interface (average score: 6.1 ± 1.3). Most participants emphasised the simplicity and intuitive nature of the interface, although some reported challenges with the device itself (e.g., keyboard and mouse functionality). These findings suggest the potential for a web-based solution compatible with mobile devices, which highlights an essential area of research as the future of cognitive assessment is likely to involve mobile applications for smartphones and tablets (43). These findings are also indicative of potential opportunities for integrating the C-STAM into routine clinical workflows, once validated through the main trial, for example as a complementary digital assessment alongside traditional screening tools or to reduce reliance on resource-intensive performance-based evaluations.

There are certain limitations to this study. The reliance on perceived subjective measures of user experience may introduce response bias, including social desirability bias, as participants may overstate positive experiences. The pilot study involved a relatively small sample of individuals with and without cognitive impairment (*n* = 30), which may limit the generalisability of the findings. Additionally, the majority of participants identified as Caucasian and had a tertiary education, which may restrict the applicability of the findings to other demographic groups, such as non-English speaking populations for whom language translation may be necessary, and individuals with limited or no prior computer experience. Furthermore, participants' prior familiarity with digital devices may have confounded usability ratings, potentially inflating scores for those comfortable with technology and underestimating challenges for less experienced users. Another limitation is the lack of objective performance metrics, such as error types, or time to complete assessments, which would complement subjective evaluations and provide a more comprehensive understanding of usability and functional engagement. These factors accentuates the importance of future studies examining cross-cultural applicability and addressing language, literacy, and computer literacy barriers and the importance of conducting larger-scale studies with more diverse populations, and some of these gaps will be addressed in the main validation study.

## 5 Conclusion

This paper presents the online implementation and initial user experience evaluation of the C-STAM, a new computerised diagnostic screening tool currently under validation, designed to assess functional ability in older adults with and without cognitive impairment. This article outlines the evolution of the design of the C-STAM, the implementation of design into practice, and the iterative feedback processes that informed its development. The user experience evaluation, conducted with 30 pilot participants with and without cognitive impairment, demonstrated a generally positive user experience and high satisfaction levels. These findings suggest that a fully computerised, self-administered assessment of functional ability is both feasible and acceptable for older adults with and without cognitive impairment. Furthermore, the principles of co-design played a critical role in ensuring that the C-STAM tool was effectively tailored to meet the needs of its target end users. The validation study will address these factors in a larger, more diverse sample to assess usability, reliability, and generalisability.

## Data Availability

The original contributions presented in the study are included in the article/Supplementary Material, further inquiries can be directed to the corresponding author.
